# Prevalence and risk factors associated with *Campylobacter* spp. and *Salmonella enterica* in livestock raised on diversified small-scale farms in California

**DOI:** 10.1017/S095026881900205X

**Published:** 2019-12-12

**Authors:** A. F. A. Pires, L. Patterson, E. A. Kukielka, P. Aminabadi, N. Navarro-Gonzalez, M. T. Jay-Russell

**Affiliations:** 1 Department of Population Health and Reproduction, School of Veterinary Medicine, University of California-Davis, Davis, CA 95616, USA; 2 Western Center for Food Safety, University of California-Davis, Davis, CA 95616, USA

**Keywords:** Agroecology, campylobacter, diversified farms, food safety, foodborne pathogens, salmonella, small-scale farms

## Abstract

Diversified farms are operations that raise a variety of crops and/or multiple species of livestock, with the goal of utilising the products of one for the growth of the other, thus fostering a sustainable cycle. This type of farming reflects consumers' increasing demand for sustainably produced, naturally raised or pasture-raised animal products that are commonly produced on diversified farms. The specific objectives of this study were to characterise diversified small-scale farms (DSSF) in California, estimate the prevalence of *Salmonella enterica* and *Campylobacter* spp. in livestock and poultry, and evaluate the association between farm- and sample-level risk factors and the prevalence of *Campylobacter* spp. on DSSF in California using a multilevel logistic model. Most participating farms were organic and raised more than one animal species. Overall *Salmonella* prevalence was 1.19% (95% confidence interval (CI_95_) 0.6–2), and overall *Campylobacter* spp. prevalence was 10.8% (CI_95_ = 9–12.9). Significant risk factors associated with *Campylobacter* spp. were farm size (odds ratio (OR)_10–50 acres: less than 10 acres_ = 6, CI_95_ = 2.11–29.8), ownership of swine (OR = 9.3, CI_95_ = 3.4–38.8) and season (OR_Spring: Coastal summer_ = 3.5, CI_95_ = 1.1–10.9; OR_Winter: Coastal summer_ = 3.23, CI_95_ = 1.4–7.4). As the number of DSSF continues to grow, evaluating risk factors and management practices that are unique to these operations will help identify risk mitigation strategies and develop outreach materials to improve the food safety of animal and vegetable products produced on DSSF.

## Introduction

Numerous alternative farming systems have emerged in recent decades as a response to the negative impacts of conventional and/or monoculture agriculture, including the encouragement of sustainable animal production [1]. Alternative livestock production systems include diverse practices such as organic, pasture-based, outdoor-raised, free-range, grass-fed, antibiotic-free, natural and sustainable practices [1–3]. This type of farming reflects consumers' increasing demand for local, sustainably grown products (including animal products labelled as naturally-raised or pasture-based, terms commonly used for sustainably-raised products from diversified small-scale farms (DSSF)) often sold at direct-to-consumer marketing venues such as community supported agriculture (CSA) and farmers markets, including animal products labelled as naturally-raised or pasture-based [2]. However, research regarding food safety risks associated with these alternative farming systems that incorporate animal production is scarce.

Diversified farms are operations that raise a variety of crops (e.g. vegetables, orchard fruit, grapes) and/or multiple species of livestock, with the goal of utilising the products of one for the growth of the other, thereby fostering a sustainable cycle [2–4]. They are also often small-scale with the intent of selling directly to consumers [2, 3]. Livestock integration on these diversified farms (also called mixed crop-livestock farms or integrated livestock farms) may be classified temporally, spatially or based upon partial or full integration of animals and crops: spatially separated (livestock and crops are physically isolated), partial integration (animals and crops occupy the same field but at different times) and fully integrated (animals graze underneath or in between crops while they grow) [3]. DSSF that integrate livestock may use their animals to graze forage crops [3], post-harvest crop residues and/or cover crops [4]. Integrating livestock and cropland provides benefits, such as reducing pests or weeds, improving soil fertility, strengthening farm income and increasing regional food security [3]. However, these types of integrated systems may contain unknown inherent risks from foodborne pathogens naturally carried by livestock, which could contaminate fresh produce crops without adequate pre-harvest mitigation practices to reduce cross-contamination risks [4]. In particular, there is a risk of pathogen contamination for farms using untreated manure and integrating livestock in fresh produce crops, which normally are eaten raw (without killing step) [5].

Some DSSF may use raw manure to improve soil quality and fertility, either directly through manure applications or through grazing of fields [5]. However, third-party auditors and regulators may discourage or prohibit integrating livestock with crops that are vulnerable to microbial contamination (e.g. leafy greens, tomatoes). Raw manure application can introduce foodborne pathogens (e.g. *Campylobacter* spp., Shiga toxin-producing *Escherichia coli* or *Salmonella enterica*) into vegetable crop fields, which could lead to contamination of produce [6]. *Campylobacter* and *Salmonella* spp. are commonly isolated from both pasture-raised livestock and poultry [7–10] and can persist in the soil for extended periods of time [2]. There is a knowledge gap regarding potential microbial risks for cross-contamination in diversified farms producing fresh produce and partially or fully integrating livestock. *Salmonella* and *Campylobacter* spp. persistence has been identified on vegetables sold at farmers markets, a common marketing venue for DSSF products [11, 12]. Moreover, the high prevalence of *Salmonella* and *Campylobacter* spp. found in organic and/or pastured-raised poultry carcasses and farm environments [7, 8] highlights the need to investigate the presence of these foodborne pathogens on California DSSF.

The aim of this study was to investigate California DSSF regarding the presence of two main foodborne pathogens: *Campylobacter* spp. and *Salmonella* spp. The specific objectives of this study were to characterise the unique attributes of DSSF in California, estimate the prevalence of *Campylobacter* spp. and *Salmonella enterica* in livestock and poultry, and evaluate the association between farm- and sample-level risk factors and the prevalence of *Campylobacter* spp. on DSSF in California.

## Materials and methods

### Study design

#### Study area and farm enrolment

Four California farming regions were selected based on their high numbers of DSSF and proximity to the UC-Davis facilities located in Yolo County. Selected regions were: Central Valley, Central Coast, North Coast Mid and West and North Interior, which corresponded to different bioregions within the state, each having different characteristics of the soil, surface water sources, landscape, wildlife and climate [13].

Farms were enrolled in this study based on the following criteria: (i) small- to medium-scale farm (i.e. poultry producers selling <1000 birds per year or livestock producers with an annual gross-sales <$500 000, and with a maximum of 500 goats/sheep, 100 cows or 100 pigs) [14]; (ii) raise a diversity of crops and/or multiple species of livestock and poultry; (iii) market their products directly to consumers (i.e. through farmers markets, CSA or other direct-to-consumer channels) and (iv) willingness to participate. Recruitment was conducted by personal invitation, phone or personal visits through various outlets (i.e. listservs, farmer associations, farmers markets) and through snowball sampling (i.e. recruited farmers recommended other farmers to be contacted) during January–April 2015.

#### Sample collection

All sampling events occurred between May 2015 and June 2016, at least twice at each farm (e.g. summer/fall and winter/spring) in order. This sampling scheme attempted to assess seasonal variations, as previous reports have described seasonality of *Salmonella* in cattle (summer/fall), swine (winter/spring) and sheep (spring), and *Campylobacter* in sheep and cattle (summer) [14–18]. This repeated cross-sectional study was part of a larger, multi-state survey that focused on small-to-medium-sized produce farms (i.e. growing leafy greens and tomatoes) and potential environmental and production factors associated with risk of microbial contamination [19, 20].

Proportional stratified sampling was conducted for each farm. Strata were based on livestock species (i.e. cattle, swine or small ruminants). The number of individual faecal samples collected varied from 1–19 per livestock species, depending upon the total count of animals per species per farm (range: 1 to 300 animals) and based on an estimated 5% *Salmonella* and *Campylobacter* spp. prevalence [21, 22] with 95% confidence and 10% error. Individual livestock faecal samples were collected from the ground in the paddocks, barn and/or pastures. Individual fresh faecal pats available in the pasture or barn were collected using gloves and brought to the laboratory within 2–3 h or shipped overnight with ice packs. Briefly, 50 g of faecal material was scooped (Bel-Art, Wayne, NJ) aseptically into a sterile sampling cup (National Scientific, Rockwood, TN) for *Salmonella* culture. Using a sterilised tongue depressor, approximately 2 g of each faecal sample was placed in a Semisolid Aerobic Enrichment Medium (SAEM) tube to transport to the laboratory for *Campylobacter* spp. isolation [23].

For poultry samples (including chickens, turkeys, geese, ducks and guinea fowl), two types of samples were collected: composite faecal samples (pool of five individual samples) and environmental swabs (drag swabs and/or surface swabs) depending on poultry flock size or number of mobile coops (i.e. poultry housing trailers). Composite faecal samples were collected as described above. Environmental samples (drag and surface swabs) were collected following the National Poultry Improvement Plan's methodology [24]. Briefly, drag swabs and swabs were made of gauze in the laboratory, autoclaved and then enriched with fat-free evaporated milk (Nestle, Solon, OH) and stored in sterile Whirlpack bags (Nasco, Modesto, CA) in the refrigerator. Drag swabs were dragged on the floor of the coops using a zig-zag pattern until the entire surface of the gauze was sufficiently covered. Surface swabs were used in areas with high concentrations of poultry faeces, including roosts, egg laying boxes and coop walls. All samples were transported in coolers containing ice packs to the laboratory within 24 h after sample collection. Processing of the samples occurred within 24–48 h of sample arrival to the laboratory.

### Environmental parameters and farmer survey

Weather data (e.g. daily temperature (°C), daily average humidity (%), daily maximum and minimum temperature (°C)) of interest were obtained from the California Irrigation Management Information System (CIMIS) based on the closest weather station for each farm and sampling date [25]. The nearest CIMIS station was determined based on distance and similar microclimate. Farmers were asked to complete a questionnaire regarding livestock health, farm demographics and biosecurity and management practices. The full questionnaire can be accessed upon request.

### Laboratory methods

#### Salmonella enterica

Upon arrival to the laboratory, 10 g of each faecal sample, or the entire swab, were placed into 90 mL Tryptic Soy Broth (TSB) (BD, Sparks, MD) and incubated at 25 °C for 2 h followed by 42 °C for 8 h, then held at 6 °C (Eppendorf, Hauppauge, NY). From this TSB enrichment, 1 mL was transferred into 9 mL Buffered Peptone Water (BPW) (Hardy Diagnostics, Santa Maria, CA) and incubated at 37 °C for 24 h. Then, 100 µL of BPW enrichment was incubated in 10 mL Rappaport Vassiliadis (RV) broth (BD, Sparks, MD) at 42 °C for 24 h followed by plating onto Xylose lysine tergitol 4 agar (XLT4) (BD, Sparks, MD) and incubation at 37 °C for 24 h [26]. Presumptive positive colonies were confirmed using traditional polymerase chain reaction (PCR) [27].

#### Thermophilic *Campylobacter* spp

The enrichment was processed according to Jeffrey *et al*. with modifications [23]. Briefly, SAEM tubes containing faecal samples were vortexed and incubated at 37 °C for 24 h. Swabs were washed in 10 mL PBS and then 5 mL of that rinsate was transferred into SAEM tubes. The enriched SAEM tubes were centrifuged at 5000 RPM for 20 min (Thermo Scientific Sorvall RC-6 plus, Ashville, NC) and 50 µL of supernatant was plated onto Campy-Cefex Agar (Acumedia, Lansing, MI) followed by incubation in anaerobic jars at 42 °C for 48 h under microaerophilic conditions (10% CO_2_, 5% O_2_ and 85% N_2_) using BD GasPak EZ container systems (BD, Sparks, MD) [28]. Presumptive thermophilic *Campylobacter* spp. positive colonies were confirmed using a real-time PCR as described previously [29]. *Campylobacter* spp positive isolates were subjected to an additional multiplex real-time PCR to identify *C. coli* and *C. jejuni* [30].

### Data analysis

For *Salmonella* data, only descriptive analyses on prevalence were conducted, due to the low number of positive samples (12/1011). Regarding *Campylobacter* spp. data, descriptive analyses were performed for continuous (e.g. median of non-normally distributed variables, inter-quartile ranges and logistic regression methods) and categorical variables (e.g. relative frequencies, percentages and χ^2^ or Fisher's exact test). Confidence intervals at the 95% level were obtained for proportions, using the Clopper and Pearson exact method from the DescTools package in R [31].

The association between farm- and sample-level risk factors and the prevalence of *Campylobacter* spp. was assessed as follows. The outcome of interest was the binary variable presence/absence of *Campylobacter* spp. (i.e. *C. coli*, *C. jejuni* and *C.* spp.) per sample. Independent variables comprised both farm- and sample-level data. At the farm-level, variables included were: years of farming; size of farm (i.e. number of acres); number of year-round employees; farming practice (‘conventional’, ‘non-certified organic’ or ‘certified organic’); ownership of cattle, small ruminants, poultry or swine; whether the farm raised more than one type of livestock; whether the farm grazed more than one livestock/poultry species on a pasture at the same time; if more than one livestock/poultry species shared the same barn at the same time; whether the farm vaccinated for any pathogen; what pathogens the farmer vaccinated against and whether the farmer sought veterinary care in the 12 months prior to the questionnaire. Farms were classified as ‘certified organic’ if they follow the USDA NOP requirements [32], and ‘non-certified organic’ farms follow organic practices but are not certified by a third-party organic certification agency. At the sample-level, variables included: season when the sample was collected (‘winter’, ‘spring’, ‘coastal summer’, ‘hot summer’ and ‘fall’; ‘hot summer’ was defined based on microclimates of inland areas and the Central Valley *vs.* cooler coastal areas), minimum and maximum temperature as well as average humidity, type of sample (‘faeces’ or ‘swab’), species of animal the sample was gathered from (‘cattle’, ‘small ruminant’, ‘poultry’ or ‘swine’) and whether the farm had more than one site.

Univariate analysis was conducted to study the distribution of values and the need for re-categorisation of variables. Variables that had little to no variation were discarded. Bivariate analysis between the outcome and each independent variable was conducted. Due to the binary nature of the outcome, we used frequency tables and χ^2^ or Fisher's exact test for each categorical or binary variable, and boxplots and simple logistic regression for each continuous variable. Variables associated with the outcome during bivariate analysis (i.e. *P*-value <0.2) were kept and included in a manual step-forward model building process leading to a multilevel logistic model with random intercept. This type of model was selected to account for clustering of data from samples collected within a farm (i.e. ‘farm’ was included as a random effect) [33, 34]. Model selection was conducted by assessing AIC values (lower values preferred), the significance of *P*-values (<0.05) and the intraclass correlation coefficient (ICC). We approximated the ICC for a random intercept logistic model using the latent variable approach [34, 35] to assess for clustering in our data [35]. A non-linear optimiser (i.e. Powell's BOBYQA method, from the lme4 package in R software) [36] was used to increase the number of iterations and improve model convergence. Multicollinearity was assessed by the variance inflation factor [37]. Biologically plausible interactions were assessed (i.e. size of farm and farming practice; size of farm and type of animal raised; and whether the farm vaccinated for any pathogen and whether the farmer sought veterinary care in the 12 months prior to the questionnaire) and considered significant at a *P*-value level of <0.20. Proxy variables of potential confounders based in causal diagrams (animal health status, age and production stage) were evaluated as well (veterinary visit in last year, production type, layer chickens *vs.* broilers). Model diagnostics consisted of the exploration of high-level residuals (level 2, i.e. farm level) by a *qq*-plot and a caterpillar plot. Statistical analyses were performed using R (version 3.3.1, R Core Team) [38].

## Results

Twenty DSSF participated in this study. Eleven farms (55%) were located in the Central Valley bioregion, four (20%) in the Central Coast, two (10%) in the North Coast Mid and West, and three (15%) in North Interior [13]. Eleven farms (55%) were certified organic, whereas five (25%) were non-certified organic and four (20%) were conventional. Four (20%) of the participating farms only raised livestock, no produce. Seventeen (85%) of the farms owned their own livestock, with four (20%) of those only raising poultry. Three produce farms leased sheep from a neighbour to graze their cover crops or crop residues during the year. Of the 16 farms that also grow produce, 56.3% (9/16) integrate livestock into produce fields to graze crop residues post-harvest and/or graze cover crops pre-planting, and 43.8% (7/16) raise livestock but keep them separate from crop fields (spatially separated per earlier terms). Farms were sampled for a median of three times (IQR = 2–3) and each farm averaged a median number of samples of 43 (IQR = 26.75–66.5) during the study period.

In total, 1011 samples were collected: 745 faeces, 153 poultry house drag swabs and 111 poultry house surface swabs. Faecal samples belonged to the following species: cattle (beef and dairy, *n* = 113), sheep (*n* = 209), swine (*n* = 143), goats (dairy, meat, and fibre, *n* = 93), chickens (broilers and layers, *n* = 181) and other poultry (*n* = 19).

### *Salmonella enterica* and *Campylobacter* spp. prevalence

The presence of *Salmonella enterica* was screened in 1011 samples, and 12 were positive for this pathogen (overall prevalence = 1.19% (CI_95_ = 0.6–2)). Percent positive samples per farm ranged from zero to 10.39%. Layer chickens, sheep and swine were found positive for *Salmonella* (prevalence = 1.27% (CI_95_ = 0.4–2.9) (5/399), 1.91% (CI_95_ = 0.5–4.8) (4/209) and 2.10% (CI_95_ = 0.4–6) (3/143), respectively). None of the samples from cattle, goats, broiler chickens and other poultry were positive. The season with the highest *Salmonella* prevalence was winter, with 3.51% (CI_95_ = 1–8.7) (4/114) of the samples being *Salmonella*-positive, followed by hot summer with 1.59% (CI_95_ = 0.6–3.3) of positive samples (7/440), and spring, with 0.5% (CI_95_ = 0.01–2.8) positives (1/200). No positive samples were found during fall or coastal summer. By type of sample, the proportion of positives was 1.96% (CI_95_ = 0.4–5.6) (3/153) for drag swabs, 1.07% (CI_95_ = 0.5–2.1) (8/747) in faeces and 0.90% (CI_95_ = 0.02–4.9) (1/111) for surface swabs.

*Campylobacter* spp. was detected in 109 out of the 1009 samples tested for this pathogen (overall prevalence = 10.8%; CI_95_ = 9–12.9). Percent positive samples per farm ranged from zero up to 29.55% of samples tested. *Campylobacter jejuni* was the species most frequently isolated (*n* = 55; 32 in layer chickens, 10 in cattle, five in small ruminants, three in broiler chickens, three in other poultry and two in swine), followed by *C. coli* (*n* = 28; 12 in small ruminants, 11 in swine and five in layer chickens) and other thermophilic *Campylobacter* species (*n* = 22). Four chicken samples (broilers and layers) were positive for both *C. coli* and *C. jejuni*. The total *Campylobacter* spp. prevalence was 15.93% (CI_95_ = 9.7–24) (18/113) in cattle, 11.25% (CI_95_ = 8.3–14.9) (44/391) in layer chickens, 15.79% (CI_95_ = 3.4–39.6) (3/19) in other poultry, 10.49% (CI_95_ = 6–16.7) (15/143) in swine, 9.76% (CI_95_ = 2.7–23.1) (4/41) in broiler chickens, 8.61% (CI_95_ = 5.2–13.3) (18/209) in sheep and 7.53% (CI_95_ = 3.1–14.9) (7/93) in goats. *Campylobacter* spp. was detected in all seasons: prevalence was 18.42% (CI_95_ = 11.8–26.8) (21/114) in winter, 16.50% (CI_95_ = 11.6–22.4) (33/200) in spring, 7.72% (CI_95_ = 5.4–10.6) in hot summer (34/440), 11.31% (CI_95_ = 6.9–17.1) (19/168) in coastal summer and 2.30% (CI_95_ = 0.3–8) (2/87) in fall. *Campylobacter* spp. was recovered from 13.02% (CI_95_ = 10.7–15.7) (97/745) of the faecal samples, 4.58% (CI_95_ = 1.9–9.2) (7/153) of the drag swabs and 4.50% (CI_95_ = 1.5–10.2) (5/111) of the surface swabs. Descriptive analysis of farm and sample characteristics stratified by *Campylobacter* spp. presence/absence is presented in Table 1. For example, we can see that medium-sized farms (10 ⩽ 50 acres) represent 22.7% of the farms that tested negative for *Campylobacter* spp., whereas they represent 36.7% of the farms that yielded a positive result for *Campylobacter* spp.

**Table 1.** Characteristics of farm, sample and environmental variables stratified by *C.* spp. status from 20 small-scale diversified California farms sampled between May 2015 and June 2016

Variable	Negative for *C.* spp *N* = 900	Positive for *C.* spp. *N* = 109	*P*-value	# of analysed samples
Years farming			0.001	1009
<10 years	537 (59.7%)	83 (76.1%)		
10 ⩽ 25 years	260 (28.9%)	24 (22.0%)		
>25 years	103 (11.4%)	2 (1.83%)		
Acres			0.001	1009
<10 acres	278 (30.9%)	20 (18.3%)		
10 ⩽ 50 acres	204 (22.7%)	40 (36.7%)		
>50 acres	418 (46.4%)	49 (45.0%)		
Year-round employees			0.048	1009
1–4	597 (66.3%)	84 (77.1%)		
10–30	88 (9.78%)	10 (9.17%)		
>30	215 (23.9%)	15 (13.8%)		
Farming practice			0.025	1009
Conventional	229 (25.4%)	36 (33.0%)		
Non-certified organic	217 (24.1%)	33 (30.3%)		
Certified organic	454 (50.4%)	40 (36.7%)		
Raise multiple animal species			0.007	1009
No	152 (16.9%)	7 (6.42%)		
Yes	748 (83.1%)	102 (93.6%)		
Owns cattle			0.138	1009
No	228 (25.3%)	20 (18.3%)		
Yes	672 (74.7%)	89 (81.7%)		
Owns small ruminants			0.192	1009
No	326 (36.2%)	47 (43.1%)		
Yes	574 (63.8%)	62 (56.9%)		
Owns poultry			0.016	1009
No	69 (7.67%)	1 (0.92%)		
Yes	831 (92.3%)	108 (99.1%)		
Owns swine			<0.001	1009
No	291 (32.3%)	14 (12.8%)		
Yes	609 (67.7%)	95 (87.2%)		
Shared pasture			0.011	953
No	301 (35.7%)	53 (48.6%)		
Yes	543 (64.3%)	56 (51.4%)		
Shared barn			0.113	953
No	714 (84.6%)	99 (90.8%)		
Yes	130 (15.4%)	10 (9.17%)		
Vaccinate			<0.001	835
No	255 (34.1%)	7 (8.05%)		
Yes	493 (65.9%)	80 (92.0%)		
Type of vaccine			0.018	573
*Salmonella*	36 (7.30%)	6 (7.50%)		
*E. coli* O157	98 (19.9%)	27 (33.8%)		
Other	359 (72.8%)	47 (58.8%)		
Veterinary care			<0.001	953
No	512 (60.7%)	45 (41.3%)		
Yes	332 (39.3%)	64 (58.7%)		
Sample season			<0.001	1009
Coastal summer	149 (16.6%)	19 (17.4%)		
Fall	85 (9.44%)	2 (1.83%)		
Hot summer	406 (45.1%)	34 (31.2%)		
Spring	167 (18.6%)	33 (30.3%)		
Winter	93 (10.3%)	21 (19.3%)		
Daily min T (°C)	11.6 (7.3–16.3)	8.7 (2.9–11.6)	<0.001	1009
Daily max T (°C)	29 (24.1–36.4)	25.3 (14.4–29.9)	<0.001	1009
Average humidity (%)	0.46 (0.46–0.46)	0.55 (0.55–0.55)	0.006	1009
Type of sample			<0.001	1009
Swab^a^	252 (28.0%)	12 (11.0%)		
Faeces	648 (72.0%)	97 (89.0%)		
Source^b^			0.157	1009
Poultry	400 (44.4%)	51 (46.8%)		
Cattle	95 (10.6%)	18 (16.5%)		
Small ruminant	277 (30.8%)	25 (22.9%)		
Swine	128 (14.2%)	15 (13.8%)		
Multisite farm			0.064	1009
Multisite	593 (65.9%)	82 (75.2%)		
One site	307 (34.1%)	27 (24.8%)		

For continuous non-normally distributed variables, results are presented as: median (IQR) and *P*-values pertaining to a logistic regression output. For categorical variables, results are presented as: relative frequencies, percentages and *P*-values pertaining to a *χ*^2^ or Fisher's exact test.

a Includes both surface and drag swabs.

b Source = species of animal the sample was gathered from; poultry includes: other poultry, layer and broiler chickens; small ruminant includes: goats and sheep.

### Risk factors associated with *Campylobacter* spp

Results of the multilevel logistic model suggest that the presence of *Campylobacter* spp. is associated with size of farm (medium sized farms (10–50 acres) had a higher risk of *Campylobacter* spp. presence than small sized farms (<10 acres) (OR_10–50 acres: less than 10 acres_ = 6, CI_95_ = 2.11–29.8)), ownership of swine (OR = 9.3, CI_95_ = 3.4–38.8) and season when the sample was collected (collecting the faecal sample during spring or winter seasons (OR_Spring: Coastal summer_ = 3.5, CI_95_ = 1.1–10.9; OR_Winter: Coastal summer_ = 3.23, CI_95_ = 1.4–7.4) was associated with a higher risk of *Campylobacter* spp. presence than collecting the faecal sample during coastal summer) (Table 2). Variation across farms regarding the outcome decreased considerably when accounting for the independent variables included in the model (i.e. ICC_null model_ = 0.26 *vs.* ICC_final model_ = 0.07; an ICC of 0.07 indicates that a 7% chance of having a positive *Campylobacter* spp. sample was explained by between-farm differences). The plausible biological interactions were either not significant or the models failed to converge. A multivariate logistic regression model was also fitted and examined. Both models suggested the same variables as risk factors.

**Table 2.** Association between the presence of *C.* spp. in faecal and environmental swab samples and risk factors collected from 20 diversified small-scale California farms between May 2015 and June 2016, as suggested by a multilevel logistic model

Variable name	Coefficient	OR	95% CI
**Intercept**	−4.92		
**Size (<10 acres)**	Reference		
**Size (10 ⩽ 50 acres)**	1.79	5.99	2.11–29.78*
**Size (>50 acres)**	−0.01	0.99	0.36–3.19
**Owns swine (no)**	Reference		
**Owns swine (yes)**	2.23	9.27	3.42–38.8*
**Sample season (coastal summer) **	Reference		
**Sample season (fall)**	−1.33	0.26	0.04–1.01
**Samples season (hot summer) **	0.13	1.14	0.35–3.52
**Sample season (spring) **	1.24	3.46	1.12–10.87*
**Sample season (winter) **	1.17	3.23	1.44–7.36*
**AIC**	626.0		
**ICC**	0.071		

*P-Value < 0.05.

## Discussion

The present study characterised 20 DSSF in California, estimated the prevalence of *Salmonella* and *Campylobacter* spp. in environmental and livestock faecal samples, and modelled the association between *Campylobacter* spp. prevalence and farm- and sample-level risk factors. Most farms in our study were organic (certified and non-certified organic) and raised more than one animal species. Overall *Salmonella* prevalence was 1.19%, whereas overall *Campylobacter* spp. prevalence was 10.8%. Significant risk factors associated with *Campylobacter* spp. were farm size (number of acres), ownership of swine and season when the sample was collected.

The relatively small *Salmonella* overall prevalence of 1.19% found in this study is similar to that reported by other studies conducted in California. Gorski *et al*. detected *Salmonella* in less than 1% of cattle faecal samples collected primarily from cow-calf ranches in the Central California Coast [39]. Roug *et al*. reported a 1.97% *Salmonella* prevalence in California county fair livestock, which are frequently raised on small-scale farms or backyard premises [40]. Likewise, Bolton *et al*., reported a relatively low overall prevalence (4.3%) on four Irish farms raising more than one livestock species, defined by the authors as ‘mixed farms' [21]. In contrast, a higher *Salmonella* prevalence (17.4%) was reported for Washington DC metropolitan and Maryland mixed crop-livestock farms (as defined by the authors, livestock and vegetables grown closely on the same premise) [11]. Differences in overall prevalence could be explained by farm management practices, species-specific factors, regional differences, farm size, seasonal patterns and/or survival of the pathogen in the environment [7, 9, 22, 39, 41].

In the present study, most DSSF were certified organic or followed organic practices. Certified organic broiler chicken farms had a lower *Salmonella* prevalence than conventional broiler farms [41]. In our study, only layer chicken samples tested positive for *Salmonella* and the proportion of positive samples was higher in faecal samples than in environmental samples. A recent California survey on pasture-raised layer hens reported that only 1 of 11 farms tested *Salmonella enterica* serotype Enteritidis positive using environmental swabs, while a higher percentage of farms (6 out of 7 unvaccinated farms) were *Salmonella* Pullorum positive on the agglutination test, indicating a high exposure to *Salmonella* spp. Group D [7]. Since most DSSF raise less than 3000 layer hens, they are exempt from the Shell Egg Food Safety Rule for surveillance and vaccination against *Salmonella enterica* serotype Enteritidis [7]. These findings suggest that there are potential food safety risks related to *Salmonella* and poultry products from pasture-raised and DSSF flocks.

The overall prevalence of *Campylobacter* spp. in this study was 10.8%. In a similar study, Salaheen *et al*. reported a prevalence of 11.16% from environmental and faecal samples collected from integrated mixed crop-livestock farms located in Maryland and Washington DC regions [12]. A much higher prevalence was found in chickens (31.9%), ducks (23.9%) and pigs (53.7%) in a study conducted on mixed farms in Vietnam [42]. In contrast, *Campylobacter* spp. was not detected in any faecal samples collected from four mixed farms in Ireland [21]. However, comparison with other studies should be made with caution due to differences in regions, study design (targeted species, farm systems and sample type) and bacteriological methods.

Campylobacteriosis continues to be a major public health concern [43]. *C. jejuni* and *C. coli* are pathogens transmitted to humans commonly through food, causing an estimated 1.3 million foodborne illness cases annually in the United States, contributing to 9% of human illnesses and 15% of hospitalisations [43]. Dairy, particularly unpasteurised milk, and poultry are usually the vehicles identified for campylobacteriosis outbreaks in the United States [44].

The present study provided a baseline prevalence estimate for *Campylobacter* spp. in livestock raised on DSSF in California. Cattle, poultry and swine were the livestock species with the highest *Campylobacter* prevalence; however, all sampled livestock species host *Campylobacter* strains that are of public health concern. *C. jejuni* was the predominant species overall, and identified mainly in poultry samples, while *C. coli* was the predominant species in swine samples. More than 65% of the DSSF in this study own more than one livestock species and 55% and 15% of all farms shared pasture and/or barn space with multiple species at the same time, respectively. The transmission between animal species and possible host-specificity should be further investigated on DSSF.

We did not test post-harvest animal products (e.g. meat and eggs), but the risk of potential *Campylobacter* spp. contamination of animal products cannot be underestimated, as one study showed that 90% of whole chicken samples sold at farmers markets in Pennsylvania were *Campylobacter* spp. positive [45]. Unpasteurised dairy and raw poultry meat have been traditionally linked to *Campylobacter* outbreaks in the US [44, 46]. *Campylobacter* was the third most common bacterial etiology reported from 2009 to 2015 in the US, causing 201 outbreaks, 2309 human illnesses, 151 hospitalisations and one death, with dairy being the main food associated [47]. In California, recent outbreaks and raw milk recalls due to *Campylobacter* contamination have been traced to pasture-based dairy farms [48, 49]; however, no outbreaks linked to DSSF have been reported. Future studies investigating the microbial profile and foodborne pathogen contamination of animal and vegetable products sold at farmers markets in California are warranted.

The present study identified significant risk factors associated with the prevalence of *Campylobacter* spp., including the season of sample collection (spring and winter more likely to be positive), farm size (10–50 acres at higher risk than farms with <10 acres) and ownership of swine (farms that own swine more likely to be positive).

A seasonal pattern in human campylobacteriosis has been reported across the globe. In the US, a strong seasonal pattern (peak in July) of human campylobacteriosis is observed, which precedes the peak of *Campylobacter* contaminated chicken carcasses. Although contaminated raw chicken is considered the leading cause of human campylobacteriosis, it might not be the primary driver for the seasonal pattern in human illness [50]. Similarly, in European countries, a pronounced seasonal human campylobacteriosis peak (mid-summer) was observed from 2006 to 2016 [51]. However, on-farm and at pre-harvest studies are in less agreement regarding the seasonality. In our study, *Campylobacter* prevalence was highest during winter and spring. Similar findings have been reported in sheep and swine in studies conducted in UK abattoirs and Canadian farms, respectively [17, 52]; in contrast, a peak prevalence during the summer was reported in cattle and sheep in a longitudinal study in UK farms [18]. A seasonal change may represent changes in management practices or suggest a relationship between changes in environmental factors (e.g. temperature and rainfall) and *Campylobacter* shedding. Several studies have identified specific risk factors associated with *Campylobacter* prevalence in livestock and poultry, including season (in sheep, swine and cattle) [10, 17, 18, 52], farm size (in cattle) [18], management practices (e.g. pasture-based systems, diet and stocking density) [10, 18, 53], environmental factors in broiler flocks [41, 54] and age (in poultry, sheep and cattle) [8, 17].

Although some studies suggest a correlation between environmental conditions and an increase of *Campylobacter* during certain seasons, climate variability across the globe is large, therefore conclusive correlations between prevalence and seasonality do not always exist, especially considering other risk factors at the farm level (e.g. flies, migratory birds or water sources) [53]. Seasonality differences may be related to higher carriage and shedding rates of *Campylobacter* during certain seasons and/or higher seasonal survival in the environment and therefore repeated exposure and transmission of the pathogen [18]. Specifically, mean temperature at the month of slaughter and rain at slaughter were associated with the presence of *Campylobacter* in broilers in a European longitudinal study [54]. *Campylobacter* spp. is a thermophilic and mesophilic bacterium, with a short survival time in the environment, and is rapidly inactivated when exposed to high temperatures or dry environments [12, 55]. This sensitivity to environmental conditions and our non-use of transportation media (i.e. SAEM) for the environmental samples that were collected may explain the lower prevalence of environmental swabs as compared to faecal samples.

The farm size was associated with the risk of *Campylobacter* prevalence (10–50 acre farms had a higher risk than farms with <10 acres). Contradictory findings have been reported regarding herd size; *Campylobacte*r prevalence was significantly higher in small dairy herds in Wisconsin [56]. In contrast, cattle from large herds are more likely to be *Campylobacter* positive in a study conducted in England and Wales [57]. However, the size of the herd was not associated with *C. jejuni* prevalence in culled cows in Ohio [58]. Several herd-level factors might contribute to these findings, such as housing system (confined *vs.* pasture-based), stocking density, access to pasture, manure storage and wildlife presence [53]. Moreover, small to medium-sized farms frequently do not segregate their animals by age, which might contribute to a higher prevalence for those farms.

Interestingly, ownership of swine was a significant risk factor for a farm having a positive *Campylobacter* sample. Swine are considered natural reservoirs of *Campylobacter* spp., with on-farm prevalence varying depending on country, production system and management practices [10, 52, 59]. Diversified farms with swine and other livestock species may be at higher risk for cross-transmission of *Campylobacter* spp. between livestock species. Boes *et al*. reported indistinguishable *C. coli* genotypes of isolates between cattle and pigs, while *C. jejuni* genotypes were not shared between cattle, pigs and poultry in mixed production herds [60]. Jensen *et al*. demonstrated identical genotypes of *Campylobacter* spp. between pigs raised outdoors and their environment and wildlife [10]. *Campylobacter* spp. was readily recovered from feral swine samples (faeces and oral cavity) collected in the Central Coast in California, a major leafy greens production region [28]. However, contact with wildlife or feral animals was not associated with *Campylobacter* spp. presence in the present study.

Risk factors identified in this study should be considered when implementing mitigation strategies to prevent cross-contamination of meat products and vegetables on those farms using untreated manure as a soil amendment and/or farms integrating crops and animals. Both *C. jejuni* and *C. coli* have been associated with human illness due to the ingestion of contaminated animal and vegetable products [44, 45]. Cross-contamination is particularly important for those farms using untreated manure as a soil amendment, as *Campylobacter* can survive at variable rates in manure faecal-pads on pasture [18], stored manure [52], composted manure [61] and soil [6], and may increase the food safety risks of products grown on DSSF.

Limitations of this study include farm selection bias and external validity, recall bias and diagnostic test characteristics. In the absence of an official list of all DSSF in California, a random selection of farms was not possible. Although we made several efforts to enroll farms representative of Californian DSSF, participation was voluntary, and therefore these farms are unlikely to represent the spectrum of all practices used on DSSF in California. Another limitation regards sampling and diagnostic tests (culture and isolation). Fresh samples were collected from the ground. Although we made concerted efforts to collect the freshest individual samples, there was always a potential for contamination of the faecal samples from the soil or environment, which might have affected the ability to isolate *Campylobacter* in faeces. Moreover, some of the prevalence estimates are very low, with large confidence intervals due to small sample sizes of some of the variable strata. Further longitudinal studies to assess temporal variation and including a large sample size should be conducted in future.

This study contributes to the research gap regarding DSSF and food safety risks. As the number of DSSF continues to grow, evaluating risk factors and management practices that are unique to these operations will help identify scale-appropriate food safety mitigation strategies and develop outreach materials to improve the food safety of animal and vegetable products produced on DSSF.
